# Novel Hydrogel-Advanced Modified Clay Nanocomposites as Possible Vehicles for Drug Delivery and Controlled Release

**DOI:** 10.3390/nano7120443

**Published:** 2017-12-13

**Authors:** Raluca Ianchis, Claudia M. Ninciuleanu, Ioana C. Gifu, Elvira Alexandrescu, Raluca Somoghi, Augusta R. Gabor, Silviu Preda, Cristina L. Nistor, Sabina Nitu, Cristian Petcu, Madalina Icriverzi, Paula E. Florian, Anca M. Roseanu

**Affiliations:** 1 National R-D Institute for Chemistry and Petrochemistry ICECHIM—Bucharest, Spl. Independentei 202, 6th district, P.O. Box 35/174, 060021 Bucharest, Romania; gifu_ioanacatalina@yahoo.com (I.C.G.); elviraalexandrescu@yahoo.com (E.A.); ralucasomoghi@yahoo.com (R.S.); ralucagabor@yahoo.com (A.R.G.); lc_nistor@yahoo.com (C.L.N.); sabina.nitu@yahoo.com (S.N.); 2 Institute of Physical Chemistry “Ilie Murgulescu”, Romanian Academy, Spl. Independentei 202, 6th district, P.O. Box 194, 060021 Bucharest, Romania; predas01@yahoo.co.uk; 3 Institute of Biochemistry of the Romanian Academy, Ligand-Receptor Interaction Department, Spl. Independentei 296, 060031 Bucharest 17, Romania; radu_mada@yahoo.co.uk (M.I.); florian_paula@yahoo.com (P.E.F.); roseanua@gmail.com (A.M.R.)

**Keywords:** hydrogel, clay, nanocomposites, biocompatibility

## Abstract

Present study refers to the synthesis of new advanced materials based on poly(methacrylic acid) (PMAA) with previously reported own advanced modified clays by edge covalent bonding. This will create the premises to obtain nanocomposite hydrogels with combined hydrophilic-hydrophobic behavior absolutely necessary for co-delivery of polar/nonpolar substances. For the synthesis, *N*,*N*’-methylenebisacrylamide was used as cross-linker and ammonium persulphate as initiator. As a consequence of the inclusion of clay into the polymer matrix and the intercalation of PMAA between the layers as well as the presence of hydrophobic interactions occurred between partners, the final hydrogel nanocomposites possessed greater swelling degrees, slower de-swelling process and enhanced mechanical properties depending on the clay type in comparison with pure hydrogel. In vitro MTS ([3-(4,5-dimethylthiazol-2-yl)-5-(3-carboxymethoxyphenyl)-2-(4-sulfophenyl)-2*H*-tetrazolium, inner salt]) colorimetric assay showed that direct exposure with PMMA-clay-based constructs did not affect cell viability and proliferation in time (24 and 48 h) on either normal or adenocarcinoma cell lines.

## 1. Introduction

Nanocomposite hydrogels were, are and will be promising materials with very high potential in a large variety of domains, especially in biomedicine [1,2,3]. Due the synergistic molecular combination of polymeric matrix and nanometric inorganic partner, these materials meet a series of requirements related with their final application, such as: high stimuli responsiveness (heat, light, magnetic fields, chemical agents and pH), mechanical strength, high drug loading, muco- or bio-adhesive properties, fast and efficient self-healing ability [4,5,6]. Among nanocomposites hydrogels, clay mineral-containing nanocomposite hydrogels possess special qualities [7] and can be used as superabsorbents, drug vehicles, tissue scaffolds, wound dressing and biosensors [8,9]. Clay nanocomposites hydrogels were developed as a response of the increasing need for multifunctional materials with superior mechanical and biological properties compared with conventional hydrogels. Clay is a hydrophilic naturally occurring inorganic mineral salt with a layered structure, cheap to purchase with reported typical thickness dimensions of platelets around 1–1.6 nm and lengths in the range about 100–400 nm in the plane of the platelets [10,11]. The structure of 2:1 phyllosilicate clay mineral is composed of 2 silicon tetrahedral (SiO_4_^4−^) sheets sandwiching a central aluminum octahedral [Al(OH)_6_^3−^] sheet [12]. Clay functionalization is mandatory in order to achieve materials with combined hydrophilic-hydrophobic behavior able to absorb polar-unpolar substances, drugs respectively. By modifying clay structure with surfactants (e.g., alkyl ammonium salts) via the cation exchange process or with silane coupling agents by sol-gel processes, organomodified clays can be synthesized [13]. Highly functionalized nanoclay can form physical, reversible and dense crosslinked structures which are effective on dissipating energy and stabilize the hydrogel network leading to enhanced mechanical strength hydrogels. These properties play a decisive role in targeted drug delivery in which hydrogel based material should reach to a specific place totally unaltered. Silicate nanoparticles encapsulated inside a hydrogel network affect its morphology and consequently the drug diffusion process reflected in the delay of drug release. Various nanocomposites based on different polymer/biopolymer matrices and clay have been studied such as: polyacrylamide-chitosan-pristine montmorillonite, maltodextrin-co-dimethylacrylamide-vinyled montmorillonite, Poly(acrylamide)-Bentonite, clay/sodium polyacrylate hydrogels, high density polyethylene-modified sodium montmorillonite, montmorillonite-epoxi nanocomposites [14,15,16,17,18,19,20,21,22,23]. 

Over the years, several research groups demonstrated that clay addition improves the mechanical properties of the nanocomposite hydrogels due to the enhancement in the stiffness of the material [10]. Superior tensile strength and modulus for PNIPAM/laponite nanocomposite was obtained by Haraguchi et al. [24] who concluded that homogenous clay dispersion is imperative for the obtaining of superior properties, inorganic clay acting as a multifunctional cross-linker. Liu et al. [25] improved the mechanical properties of *N*-isopropylacrylamide by introducing Laponite XLG or XLS. Fukasawa et al. [26] tested the mechanical properties of a Tetra-PEG/Clay Network Nanocomposite Gel and observed that the tensile properties were enhanced when clay was added. Chang et al. [27] developed a PEG/clay nanocomposite hydrogel and demonstrated that clay addition significantly enhanced the compressive and tensile properties of the polymer matrix proposed for tissue engineering scaffold. Other group proved that the strength of clay systems was higher than free gels and the shape of nanocomposites were preserved when large stress and deformation were applied [28]. In 2014, Shen et al. studied the rheology and adhesion capacity of Poly(acrylic acid)/Laponite and showed that clay influenced fluidity and adhesion, the obtained nanocomposite hydrogels being proper candidates for bioadhesives [29]. Another study evidenced that clay improves the tensile strength, elongation and energy at break due to the strong electrostatic interaction between chitosan and clay [19]. Recently, Xu and Gersappe compared nanofillers of different shapes and demonstrated that disk-like nanofillers strengthen the hydrogels more effective than spherical nanofillers [30].

Besides enhanced thermo-mechanical properties, clays were proved to increase water retention capacity and consequently drug loading of hydrogel nanocomposites [31,32]. Nagahama group used clay nanodiks to obtain a new hybrid gel for focal cancer therapy demonstrating the efficiency of a single injection which provided long-term sustained antitumor activity [33]. Furthermore, clay presence allowed the control and modulation of drug release profiles for the nanohybrids obtained by electrostatic LbL self-assembly approach of cationic poly(allylamine) hydrochloride and anionic poly(sodiumstyrene sulfonate) [34]. Same findings were for poly (vinyl alcohol)-chitosan-Laponite RD nanocomposite hydrogels synthesized by Oliveira et al. [35].

As mentioned above, most studies use commercial Laponite or montmorillonite to prepare hydrogel nanocomposites. Moreover, researches referring to the synthesis of own advanced organomodified clays nanocomposites are just a few [21,22,23] and as far as we know just related to the inclusion of functionalized clay in hydrophobic matrices. Based on the mentioned observations, the aim of this work was to synthesize novel advanced materials based on poly(methacrylic acid) (PMAA) with in house modified clays. Starting from commercial organomodified clay, namely Cloisite 93A (Cl 93A) (methyl, dihydrogenatedtallow)-(Cloisite^®^ 93A; 90 meg/100 g), we previously reported the preparation of advanced functionalized clay by covalently bonding octyl (C8) or octadecyl (C18) alkyl chains at clay edges [36]. Our present study follows the effect of the modified clay on the physical-chemical properties of the novel hydrogels. The biocompatibility of the newly nanocomposites was also investigated in vitro. The desired materials to be obtained are foreseen to become novel bio agent carriers with enhanced mechanical properties. Moreover, by introducing advanced hydrophobic modified clays into PMAA matrix, we created the premises to obtain novel nanocomposite hydrogel structures with combined hydrophilic-hydrophobic behavior necessary for sustained release of co-entrapped polar/unpolar substances. These novel systems are envisaged for possible application in gastrointestinal cancer therapy.

## 2. Results

### 2.1. Hydrogel Nanocomposites Characterization

#### 2.1.1. Swelling/De-Swelling Properties

**Figure 1:**
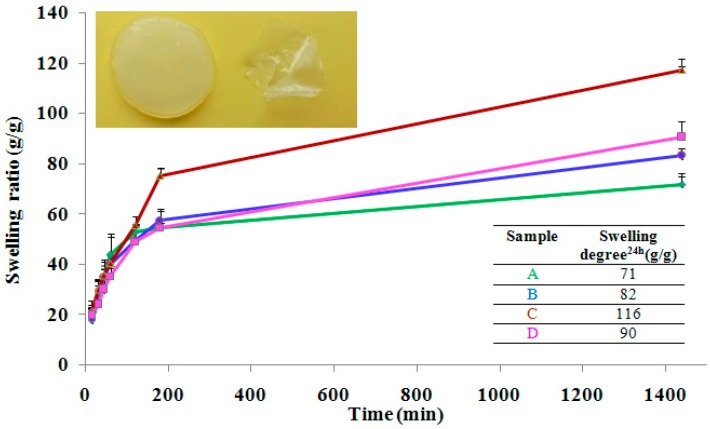
Time-dependent swelling profiles of the hydrogels and appearance of the hydrogels, dried and swollen state (~99% water); A-PMAA; B-PMAA-Cl 93A; C-PMAA-Cl 93A-C8; D-PMAA-Cl 93A-C18.

To reach swelling, the hydrogels were immersed in deionized water, replacing water several times. The hydrogel volume changed during the course of swelling. Swelling experiments demonstrated that the nanocomposite hydrogels preserved the shape and did not disintegrate in 3 months. Time-dependent swelling profiles of the hydrogels in de-ionized water were summarized in Figure 1. It was found that all hydrogels swelled rapidly in the first 3 h, followed a path of steady growth swelling and after 24 h still retain water. The swelling was faster for the blank sample—the hydrogel without clay, while for the nanocomposite hydrogels swelling was lowered very probably because was hindered by clay layers. We notice that after 100 min the curves begin to differentiate depending on the composition namely, clay samples retain more water than the blank sample. These results are in good agreement with other studies which proved that the addition of clay into polymer matrix increase the swelling degree [31,32]. After 24 h we noticed that all of the hydrogels exhibited the same trend. This fact can be due the presence of –OH groups from clay structure that attract water molecules which penetrate inside hydrogel networks. Moreover, hydrogen bonds between clay edges and polymer matrix could be established and thus modifying the diffusion of water molecules inside the complexed crosslinked structure [24,31]. In our experiments, the hydrogels obtained in the presence of in house modified clay with octyl dimethylmethoxysilane (Cl 93A-C8) and in house modified clay with octadecyl dimethylmethoxysilane (Cl 93A-C18) reached the highest degrees of swelling, compared with the blank sample the swelling degree increasing with 27% and 63%, respectively. A possible explanation of these findings could be related to the hydrogel pore dimensions and their (see the SEM section).

**Figure 2:**
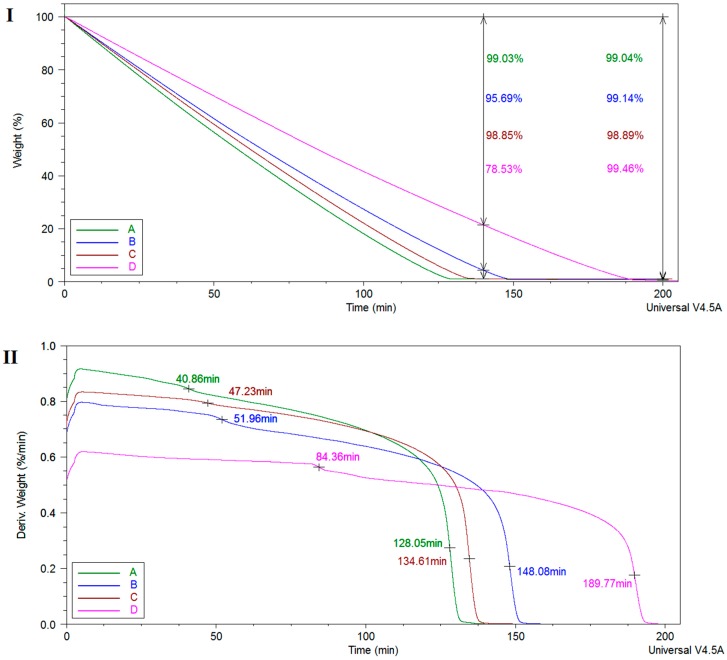
(**I**) Weight loss as function of time measured at constant temperature (37 °C) of the equilibrium swollen hydrogel samples; (**II**) Weight derivative as function of time for the equilibrium swollen hydrogel samples; A-PMAA; B-PMAA-Cl 93A; C-PMAA-Cl 93A-C8; D-PMAA-Cl 93A-C18.

The de-swelling behavior of the equilibrium swollen hydrogel samples was investigated by TGA/DTG analyses. As shown in Figure 2, derivative curves revealed a slower weight change over time (50–80 min interval) in the following order: PMAA-(PMAA-Cl 93A)-(PMAA-Cl 93A-C8)-(PMAA-Cl 93A-C18). The samples with clay exhibit slower dehydration rate and released less water compared to the pure hydrogel 99.03% water released vs. 95.69% (Cl 93A sample), 98.85% (Cl 93A-C8 sample) and 78.53% (Cl 93A-C18 sample) after 140 min. 

The collapse time is shifted toward higher values when adding clays, specifically, from 128 min to 148 min for PMAA hydrogel, 134 min and 189 min for the clay containing samples. The obtained data points indicate a water restricting capacity due to the presence of clay layers which behaved as a barrier in the water outflow from the hydrogel networks. Similar behavior was also reported observed by others and recommends these nanocomposite hydrogels for situations where controlled release of the drug is required [31,35]. After 200 min the hydrated samples lost ~99% water, as follows: [PMAA/99.04%]-[(PMAA-Cl 93A)/99.14%]-[(PMAA-Cl 93A-C8)/98.89%]-[(PMAA-Cl 93A-C18)/99.46%].

#### 2.1.2. FTIR Spectroscopy

**Figure 3:**
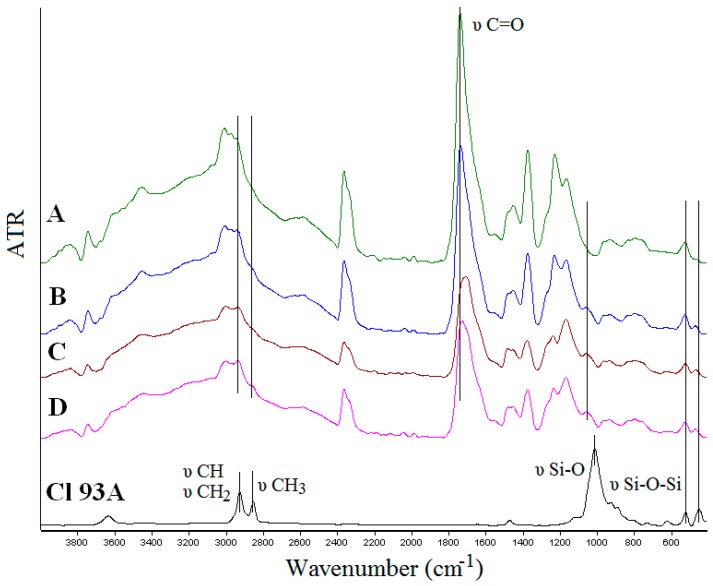
FTIR spectra of nanocomposite hydrogels: A-PMAA; B-PMAA-Cl 93A; C-PMAA-Cl 93A-C8; D-PMAA-Cl 93A-C18 and commercial clay-Cl 93A.

FTIR spectroscopy was used to prove the inclusion of clay in the PMAA matrix and to observe if interactions between the components occurred. FTIR spectra of the nanocomposites hydrogels are presented in Figure 3. Spectra of neat polymer hydrogel network, PMAA respectively, showed characteristic peaks: 1736 cm^−1^ C=O stretching vibration of carbonyl group; 1225 cm^−1^ corresponding to the carboxyl (C–O) stretching; 2942 cm^−1^ due to CH_2_ stretching; 3455 cm^−1^ attributed to the hydroxyl group (O–H) stretching absorption [37,38,39]. The absence of the characteristic signal at 1637 cm^−1^ ascribed to vinyl monomers (C=C stretching) in the spectra of PMAA, it is an evidence of its successful polymerization [40]. According to FTIR spectra of hydrogel nanocomposites, the typical peaks of Cl 93A, were found, as follow: 2849 cm^−1^ (tertiary CH and secondary CH_2_) and 2923 cm^−1^ (primary CH_3_) characteristic for quaternary ammonium salts [19,41], 441 cm^−1^ (Si–O stretching) and 516 cm^−1^ (Si–O–Si and Al–O–Si deformation) [42,43]. It has to be mentioned that the peaks corresponding to –CH_2_ and –CH_3_ groups were more intense in the case of the hydrogels prepared with advanced functionalized clay due to the presence of C8 and C18 alkyl chains from the functionalization agents [36]. Several modifications must be mentioned, as follow:

- shifted wavenumbers values around 294 cm^−1^ due to CH_2_ stretching from PMAA and quaternary ammonium salts from clays;
- shifted wavenumber values of C=O group (1736 cm^−1^) originated from PMAA toward lower values (Cl 93A—1733 cm^−1^; Cl 93A-C8—1723 cm^−1^; Cl 93A-C18—1707 cm^−1^);
- the peak of Cl 93A clay from 1007 cm^−1^ corresponding to Si–O stretching vibration, shifted toward higher values with the inclusion of clay in the PMAA matrix (1036 cm^−1^);
- clay peaks observed at 516 cm^−1^ and 441 cm^−1^ (Si–O–Si and Al–O–Si deformation) appeared in the nanocomposites FTIR spectra at higher values 518 cm^−1^ and 464 cm^−1^.

These could indicate the possible formation of hydrogen bonding between the polymer PMAA hydroxyl moiety (deformation of carbonyl group) and the clays [19,39]. Present observation led to the conclusion not only that the clays were entrapped in the hydrogel network but also clay layers interacted with the PMAA network creating a compacted/consistent complex structure.

#### 2.1.3. X-ray Diffractograms

**Figure 4:**
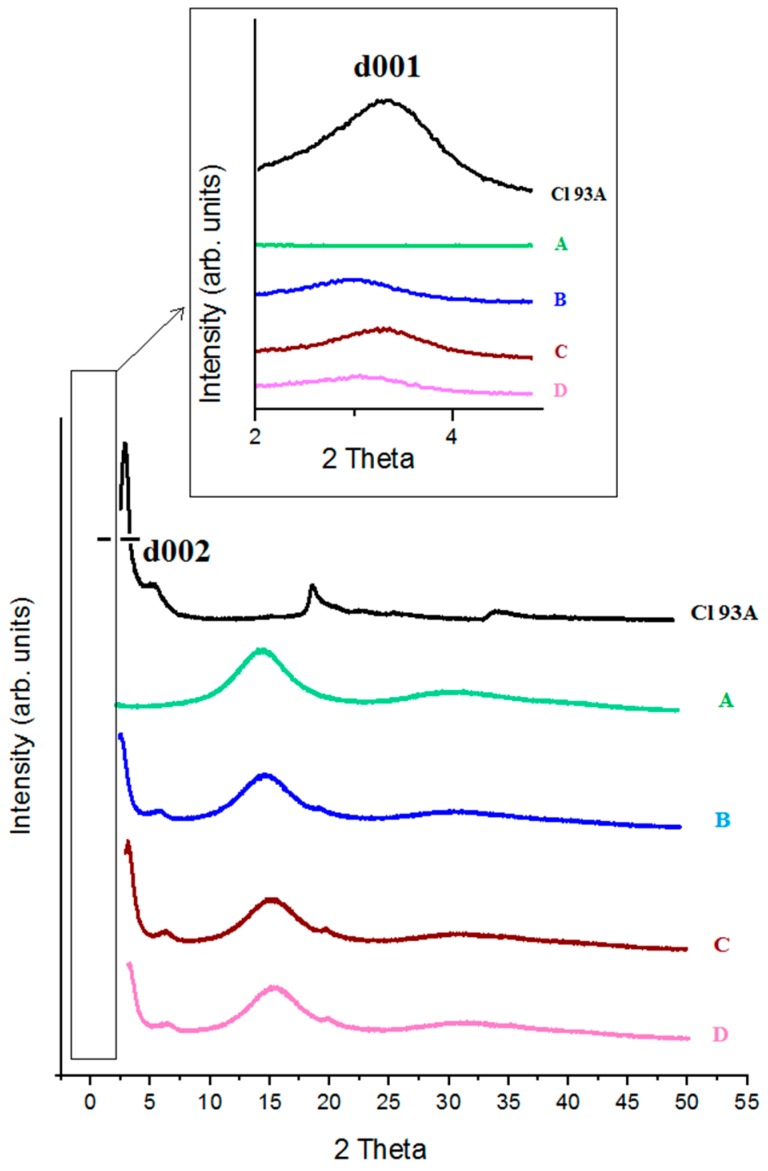
X-ray diffractograms registered for Cl 93A, PMAA (**A**), PMAA-Cl 93A (**B**), PMAA-Cl 93A-C8 (**C**), PMAA-Cl 93A-C18 (**D**); inset-Low angle XRD.

X-ray diffractograms (XRD) obtained for pure PMAA, Cl 93A and PMAA-Cl 93A, PMAA-Cl 93A-C8, PMAA-Cl 93A-C18 nanocomposites are displayed in Figure 4. In the case of PMAA we can notice a peak at 2θ = 15°, suggesting the high crystallinity of the compound [37,44]. As we expected, this peak appears also in the nanocomposites samples. 

The XRD pattern of Cl 93A shows two characteristic peaks of clays at 2θ = 3.28° and 6.55° corresponding to a basal spacing of 26.88 nm and 13.48 nm respectively (Table 1). According to the Bragg law, increasing of d-spacing results to the broadening and shifting of related diffraction line toward lower diffraction angles (2θ). By monitoring the position (2θ), shape and intensity of the characteristic diffraction line for clay in nanocomposite structure it is possible to observe the intercalation/exfoliation phenomenon [45]. When Cl 93A, Cl 93A-C8 and Cl 93A-C18 were added to the PMAA hydrogels d001 and d002 shifted to lower values which means that d-spacing increased. This phenomenon suggests that the layered silicates were mainly intercalated within the PMAA matrix, reported by others [42,46]. This finding is more accentuated in the case of PMAA-Cl 93A sample, signifying that commercial clay is more compatible with the polymer matrix, while modified clay due to the hydrophobic groups hinders the dispersion within PMAA networks.

#### 2.1.4. Thermo-Mechanical Characteristics

**Figure 5:**
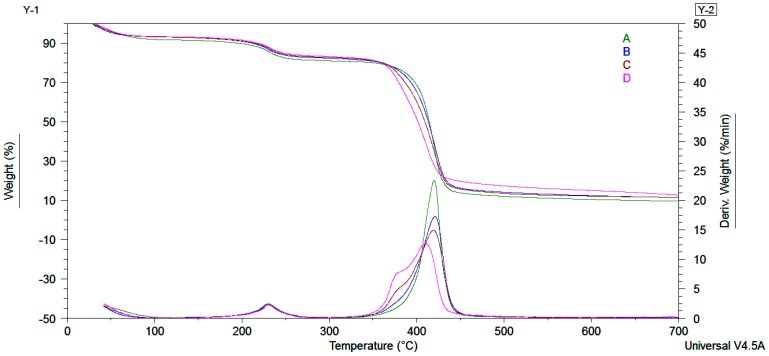
TGA/DTG curves for the obtained hydrogel nanocomposites (B-PMAA-Cl 93A, C-PMAA-Cl 93A-C8, D-PMAA-Cl 93A-C18) against polymer matrix (A-PMAA).

Thermal stability of the obtained hydrogel and hydrogel nanocomposites were examined by thermogravimetric analyses (TGA). The results generated from TGA/DTG and dynamic mechanical analyses (DMA) curves are summarized in Table 2. TGA curves showed three steps of weight loss as observed from Figure 5 and Table 2. The first step corresponds to the volatilization of water and organic compounds. The second and third steps with two maximum points at 230 °C and 480 °C from the derivative curves could be assigned to PMAA decomposition [37,47,48,49,50,51,52]. In the case of the hydrogel nanocomposites obtained in the presence of clay modified by us, a shoulder coupled with the maximum decomposition peak at 380 °C can be observed. This shoulder is correlated to the decomposition of the alkyl chains from organomodified clay structure, indirectly confirming the covalent bonding on the Cl 93A edges [36]. The residue growth is related with the presence of inorganic filler and proves the insertion of clay into PMAA hydrogels [36,43]. TGA indicated that upon inclusion of clay, thermal stability of final nanocomposite materials suffered minimal changes related to the organic part brought by the in house modified clays.

**Figure 6:**
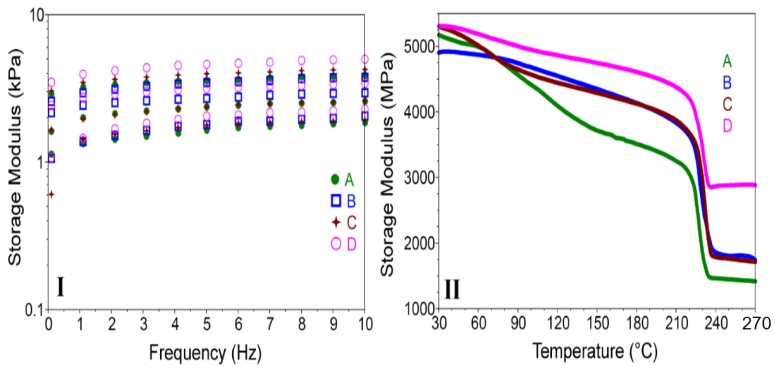
DMA results registered for pure PMAA (**A**) and PMAA-Cl 93A (**B**), PMAA-Cl 93A-C8 (**C**), PMAA-Cl 93A-C18 (**D**) nanocomposites; Storage modulus as function of: **I**. Frequency; **II**. Temperature.

Dynamic mechanical analyses (DMA) were performed in order to determine if the addition of clays affected the mechanical behavior of the obtained nanocomposite hydrogels. These characteristics play a very important role for the carrier material to reach the target situs unchanged. Storage Modulus-G’ as function of frequency of crosslinked hydrogels for twelve cycles were registered but only 2, 6 and 10 cycles were represented in Figure 6I. It can be noticed that the addition of inorganic filler increased the storage modulus when frequency was increased for all types of clay. This phenomenon could be related to strong interactions between the hydrogel and high aspect ratio clay platelets, as mentioned by other groups [53,54,55]. The storage modulus results showed that among the nanocomposite hydrogels, PMAA-Cl 93A-C18 had the highest value with increasing frequency, for all cycles registered. The G’ lowest values for cycle 2 and 6, was obtained for PMAA and PMAA-Cl 93A-C8 hydrogels. These data indicated a hindered water transport from the sample obtained in the presence of Cl 93A-C18. This is possibly the result of the hydrophobicity induced by the long alkyl chains reflected in strong hydrophobic interactions. For cycle 10, the order changed and the lowest value was obtained for PMAA-Cl 93A followed by PMAA hydrogel. These data reveal an easier release of water retained by the mentioned hydrogels compared to the samples where advanced modified clays were used. Here also, it seems that the advanced modified clays proposed by us restrict the release process and the capacity of crosslinked PMAA networks to expel the water. These facts can be correlated with FTIR analyses that indicated that hydrogel-clay complex structures were formed through hydrogen bonding interactions between the oxygen atoms of PMAA and the amine protons of the clay as well as between the clay surface hydroxyls and the carbonyl of the polymer.

In order to reach for equilibrium, the hydrogel polymer chains dynamically adsorb/desorb on/from the clay particles and consequently an extensive rearrangement of whole system occurs. These reconfigurations try to counterbalance the force acting on the composite hydrogel and thus enhanced mechanical properties are obtained [56,57]. It should be noted that, from the DMA curves allure, a good stability of the nanocomposites samples could be observed when increasing frequency. This fact is very important when the final material is subjected to mechanical demands and is beneficial to the enhanced toughness of hydrogels based on the energy dissipation mechanism [58,59].

The presence of inorganic filler increased the storage modulus of freeze-dried samples when temperature was raised (Figure 6II). For all the samples, transitions around 110 °C and 230 °C were observed. This fact is due the movement of methacrylic acid units, glass transition temperatures depending on the molecular weight.

DMA curves showed that with clay addition the mobility of hydrogel network is restricted. This behavior is supported by the increase of the glass transition temperature values for all clay types (Table 2) [60]. Among them, the highest glass transition temperature was registered for the hydrogel obtained in the presence of in house advanced modified nanoclay Cl 93A-C8/C18. Here also, long alkyl chain induced a reinforcing effect more pronounced than the commercial nanoclay type when the nancomposite hydrogel is subjected to the temperature changes [36]. In spite clay aggregation and sedimentation phenomenon, improvement of the mechanical properties induced by frequency/temperature changes of the nanocomposite hydrogels, were registered. These are generally ascribed to the clay barriers properties which slow down the movement of polymer chains [36,61,62]. Future investigations include the high-level dispersion of clay in order to obtain homogenous nanocomposite hydrogels with enhanced reinforcing effect.

#### 2.1.5. Microscopy Analyses

**Figure 7:**
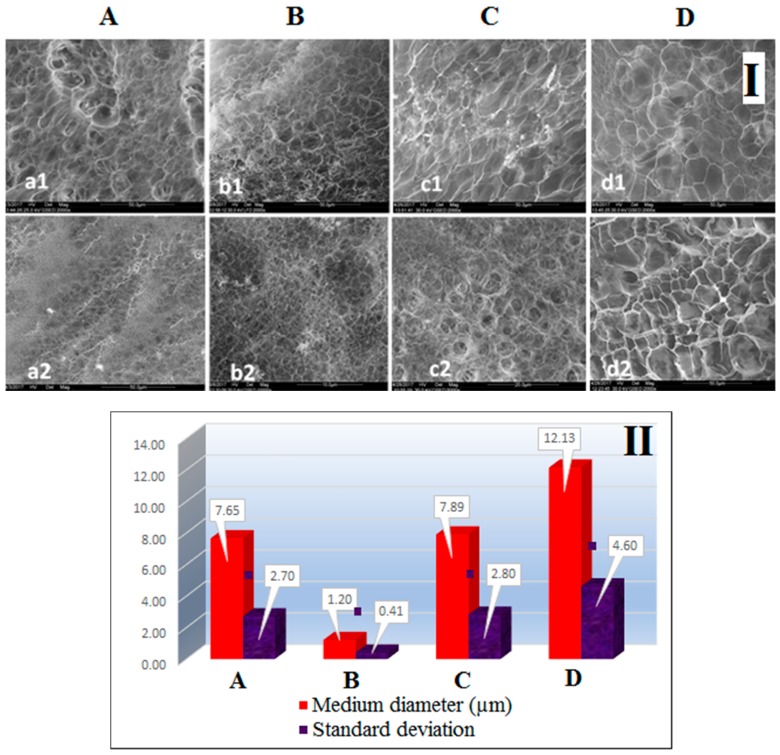
(**I**) SEM Images showing the microstructure aspect on fracture and surface of pure PMAA (**A**) and PMAA-Cl 93A (**B**), PMAA-Cl 93A-C8 (**C**), PMAA-Cl 93A-C18 (**D**) nanocomposites; a1-sample A-fracture (×2000); a2-sample A-surface (×2000); b1-sample B-fracture (×2000); b2-sample B-surface (×10,000); c1-sample C-surface (×2000); c2-sample C-surface (×5000); d1-sample D-fracture (×2000); d2-sample D-surface (×2000); (**II**) The fineness of the structure, expressed by the mean pore diameter and uniformity, estimated by the standard deviation from the mean.

SEM analyses of the freeze-dried nanocomposite hydrogels were performed to observe the morphology of the hydrogels prepared with various types of clay (Figure 7). The microstructure observed both on the examined surfaces and on the breaking surfaces, consists of mainly eclaxic voids separated by thin walls. The dimensions of these gaps vary depending on the type of clay. The finest structure with the highest level of uniformity was observed on the sample containing Cl 93A (sample B). The pore size and degree of uniformity of C sample, containing C8 modified clay, is comparable to the blank sample (pure PMAA). In addition, fine-particle, bright contrast particles, especially present in the fracture, were observed on sample C. The same particles were also observed on sample D (clay modified with C18), a sample presenting somewhat larger pores than other hydrogel-clay samples. 

The fineness of the structure, expressed by the mean of pore diameter and uniformity, can be visualized comparatively for the four samples, in Figure 7II. Interestingly, the average pore size of the hydrogels decreased when commercial clay was used for synthesis, most probably due to –OH unblocked groups from commercial clay structure which induced a higher cross-linking density and concomitant smaller pore size. Meanwhile, the clay modified by us generated greater pore sizes, although the presence of smaller pores is observed in the high pores, possible as a result of clay aggregation phenomenon [36] which disrupted the continuity of the hydrogel microstructure causing wall cracks affecting also swelling/de-swelling studies. Moreover, the results are strongly related to the clay dispersion capacity within hydrogel matrix [19,24,30] respectively with the clay modifiers structure—alkyl chain length and degree of functionalization—which led the complex structure of the final nanocomposites, as observed before in our studies [36,63].

**Figure 8:**
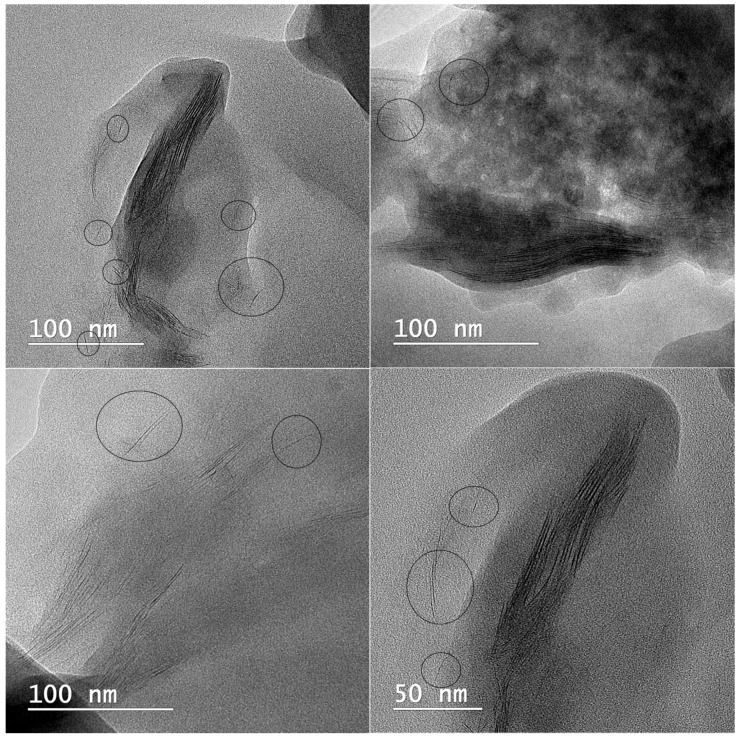
TEM micrographs showing the intercalated/exfoliated state of Cl 93A-C18 clay layers within PMAA matrix; exfoliated state is in minority and is highlighted with encirclement.

TEM analyses were used to observe the intercalated or exfoliated structure of clay inside poly(methacrylic) hydrogels. TEM images of PMAA-Cl 93A-C18 nanocomposites (Figure 8) revealed gray area representing the polymer matrix and dark lines ascribed generally for clay layers. The intercalated state, which was confirmed also by XRD measurements, can be visualized on TEM images as stacked clay layers within the polymer. Moreover, even if intercalated state prevailed, some exfoliated clay layers could be also observed. This fact can affect the mechanical properties of the final nanocomposites [36,64,65] and supports the enhanced mechanical behavior of PMAA-clay loaded nanocomposites, as proved by DMA analyses. 

### 2.2. Cell Proliferation Assay

#### 2.2.1. Cytotoxicity

**Figure 9:**
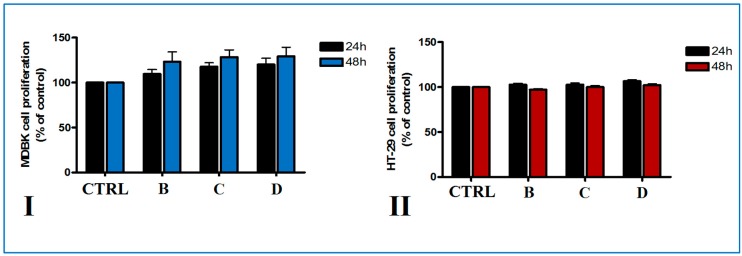
In vitro viability of MDBK (**I**) and HT-29 (**II**) cells grown in direct contact for 24 and 48 h with biopolymer-modified clays (B-PMAA-Cl 93A, C-PMAA-Cl 93A-C8, D-PMAA-Cl 93A-C18). Cell viability was expressed as percent of control (untreated cells) ± SD of three different samples.

Cytotoxicity of biopolymer modified clays on different cell lines was assessed using a MTS method. Cell viability of normal Madin-Darby bovine kidney (MDBK) cells and HT-29 colorectal adenocarcinoma cells, two in vitro model cellular lines, was measured following treatment with different disk composite for 24 and 48 h. Metabolic activity of normal and tumoral cells after the two time points of direct treatment with biopolymer-modified clays was not affected (Figure 9). Thus, no citotoxicity was observed on MDBK cells after biopolymer-modified clay disks treatment for 24 and 48 h (Figure 9I). Cell proliferation was increased compared to control the highest values of cell proliferation being registered for PMAA-Cl 93A-C18 disks, irrespective of the time of incubation. In the case of tumoral HT-29 cells, no significant difference in cell viability after the addition of disks compared to control was observed (Figure 9II). Although the unmodified Cloisite 93 A was found to be slightly cytotoxic to HepG2 cells, in our study the modified PMAA-Cl 93A-C8 and PMAA-Cl 93A-C18 did not affect the normal and adenocarcinoma cells viability. These could be as a result of chemical modification and clay entrapment into PMAA [66].

#### 2.2.2. Cell Morphology

**Figure 10:**
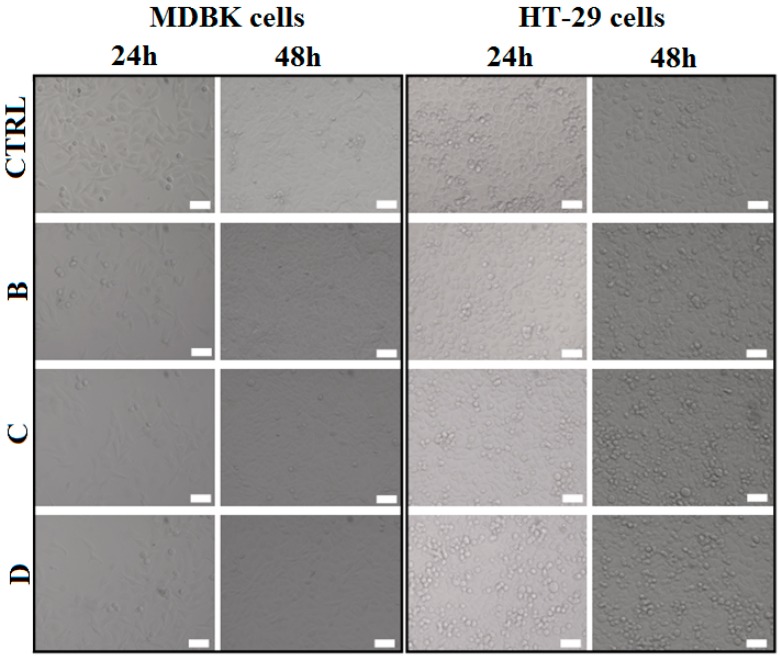
Brightfield images (20×) obtained with automated live microscopy imaging system of MDBK and HT-29 cells incubated for 24 and 48 h with or without different biopolymer-modified clays (B-PMAA-Cl 93A, C-PMAA-Cl 93A-C8, D-PMAA-Cl 93A-C18). Scale bar 50 μm.

The morphology of the cell after biopolymer-modified clay disks treatment was analyzed using a brightfield microscopy system which scanned the entire probes. As seen in Figure 10, no morphological changes were revealed in any of the cell line tested and a time dependent proliferation can be observed. The method confirmed the results obtained by MTS assay that biopolymer-modified clays did not affect cell viability and proliferation after 24 and 48 h of direct exposure on either cell lines.

## 3. Experimental Section

### 3.1. Materials

Commercial clay Cl 93A was kindly offered by Southern Clay Products Inc. (Gonzales, TX, USA) and was used as organomodified compound with different ammonium salts (methyl, tallow, bis-2-hidroxyethyl (methyl, dihydrogenatedtallow)-(Cloisite^®^ 93A; 90 meg/100 g)). The synthesis of in house modified clay with octyl dimethylmethoxysilane (Cl 93A-C8) or octadecyl dimethylmethoxysilane (Cl 93A-C18) was described elsewhere [24]. PMAA (polymethacrylic acid, Janssen Chimica), *N*,*N*′-methylenebisacrylamide (Sigma Aldrich) (Saint Louis, Mo, USA) and ammonium persulfate (Sigma Aldrich) (Saint Louis, Mo, USA) were used as received.

### 3.2. The Synthesis of Composite Hydrogels

Acrylic hydrogels containing clay were synthesized according to an adapted method [37], as follows: primarily, 0.15 g clay (Cl 93A, Cl 93A-C8, Cl 93A-C18 [36]) was dispersed in 10.5 mL water under magnetic stirring at 800 rpm at ambient temperature for 15 h followed by ultrasonication for 10 min. After cooling the dispersion, 1.5 mL methacrylic acid (MAA) and 1.5 mL *N*,*N*’-methylenebisacrylamide (BIS) (both 1%, *w*/*v*) were added. The system was kept under mechanical stirring and nitrogen atmosphere for 30 min followed by 5 min ultrasonication in an ice bath. 1.5 mL ammonium persulfate (APS) (1.2%, *w*/*v*) was added under magnetically stirring and the initial polymerization system was injected into own construction glass mold. Further, the mold was introduced into a thermostated water bath at 70 °C for 6 h. The obtained hydrogels were cut into pieces and immersed into deionized water for 10 days. The water was changed to times a day to remove the residual monomers. Finally, the hydrogels were cut with eyelet punch and a part were deposed on a polyethylene foils for water evaporation at room temperature for several days and the other part was freeze dried.

### 3.3. Hydrogel Nanocomposites Characterization

#### 3.3.1. Physical-Chemical Characterization

Samples for swelling tests were freeze-dried under vacuum at −15 °C for 2 days. Swelling tests were conducted by immersing the freeze-dried samples in deionized water at 25 °C for a period of time to reach the equilibrium state. The swelling ratio (SR) and the swelling degree (SD) were calculated using Equations (1) and (2), respectively:

SR = Wh/Wi(1)

SD = (Wh − Wi)/Wi(2)

where, Wh is the weight of hydrated hydrogel after a certain time and Wi is the weight of dried hydrogel. All experiments were performed in triplicate.

FT-IR spectra were obtained using a Fourier transform infrared spectrophotometer (Tensor 37 from Bruker, Woodstock, NY, USA), equipped with ATR Golden Gate unite, in the range 400–4000 cm^−1^. Before analyses, the air-dried samples were milled. 

The structure of the samples was determined using an X-ray diffractometer (Rigaku Ultima IV, Tokyo, Japan) with CuKα radiation (λ = 1.5406 Å), operated at 40 kV and 30 mA. The equipment was set in parallel beam geometry system and the measurements were performed in the continuous mode, at room temperature and atmospheric pressure. The equipment was set in two configurations: 1. For the wide angle range, with DS (divergence slit) = 1°, SS and RS (scattering and receiving slits) = open and receiving side Soller slit 0.5°, collecting data over the 2θ range 3–50°, with a step width of 0.02° and a scanning speed of 2°/min; 2. For the low angle range, with DS and SS (divergence ans scattering slits) = 1° and RS (receiving slits) = 0.2° and receiving side Soller slit 0.5°, collecting data over the 2θ range 0.6–6°, with a step width of 0.02° and a scanning speed of 1°/min. The samples were measured as-received, as a powder material. The thickness of the repeated units in a regular multilayer structure contained of one layer and one inter layer space is called d-spacing (d001) or basal spacing. The basal spacing of clays can be calculated from their X-ray diffractograms. The diffraction line is indicative of the d-spacing in clay structure. Using the diffraction line position (2θ) in the XRD pattern the inter-layer space can be calculated utilizing Bragg’s law, nλ = 2dsinθ, where n = an integer, λ = wavelength of X-ray radiation used in the diffraction experiments, d = the space between layers in the clay lattice and θ = measured diffraction angle [67].

Thermogravimetric measurements (TGA) were performed with Thermogravimetric Analyser TGA Q5000IR (TA Instruments, New Castle, DE, USA). The samples were heated in the following conditions: 10 °C/min, 40 mL/min, N_2_. The isotherms of equilibrium swollen hydrogels discs were performed at 37 °C, 10 mL/min, N_2_. All experiments were performed in triplicate. 

Dynamic mechanical analysis (DMA) was performed using DMA Q800 TA Instruments, (TA Instruments, New Castle, DE, USA) apparatus with Compression clamp and Dual Cantilever- POWDER CLAMP. DMA was used in two different modes, DMA Multi-Frequency—Strain—“Frequency sweep-isothermal” for hydrogels and “Temperature Ramp” for freeze-dried hydrogels. The initial test performed was a frequency sweep from 0.1 to 10 Hz at 35.00 °C with 4 µm oscillation amplitude for 50 min (Frequency sweep segment repeat for 16 times). A 12.5 mm sample holder disk is used for all the samples. A 0.01 N compressive static force was applied to the specimen to ensure that the data collected was repeatable. This also ensured that the upper compression platen did not lose contact with the hydrogel sample. Upon completion of data acquisition, the DMA software calculates the storage modulus for the sample and exports the data for plotting as a function of frequency. The second test performed was temperature ramp with 3.00 °C/min from room temperature to 275.00 °C for 35.0000, 12.7700, 3.1900 mm sample size (powder clamp-freeze-dried hydrogels) with 20 µm oscillation amplitude, 1 Hz frequency and 0.5 sampling.

For SEM analyses, the lyophilized samples were broken manually for a better observation inside the sample. The broken tiles were analyzed by environmental scanning electron microscopy (ESEM-FEI Quanta 200, Eindhoven, The Netherlands). For the obtaining of secondary electrons images, a GSED detector was used in the following conditions: pressure of 2 torr and 25–30 KV accelerating voltage. The average whole diameter was evaluated using the Scandium software after measuring 60 pores.

The morphologies of clay nanocomposites were obtained by transmission electron microscopy using TEM, Tecnai™ G2 F20 TWIN Cryo-TEM, FEI Company™ (Eindhoven, The Netherlands), at 200 kV acceleration voltages. Samples were embedded in epoxy matrix and cut in thin sections (~100 nm) at room temperature, using a Leica EM UC7 ultramicrotome (Eindhoven, The Netherlands) and then deposited on carbon film grids.

#### 3.3.2. In Vitro Cell Assay

●  Cell culture

The MDBK cell line (Madin-Darby bovine kidney) obtained from ECACC (European Collection of Animal Cell Culture, Porton Down, UK) and HT-29 human colorectal adenocarcinoma cell line (a kind gift from Frank-Dietmar Böhmer, Institute of Molecular Cell Biology, Medical Faculty Friedrich-Schiller University, Jena, Germany) were maintained in RPMI 1640 medium and DMEM medium respectively, with stable glutamine and supplemented with 10% fetal bovine serum and 100 U/mL penicillin, 100 μg/mL streptomycin (all from Biochrom, Berlin, Germany). Cells were incubated at 37 °C in a humidified atmosphere with 5% CO_2_. Cell viability was determined by trypan blue dye exclusion and cells were used on more than 90% viability. The number of cell passage used in this work varied between 8 and 10.

●  Sterilization

Before any biological in vitro experiments, the disks were sterilized using ethanol method. Briefly the disks were immersed in 96 well plates for 2 min in 70% ethanol solution prepared in PBS (phosphate buffered saline at pH 7.4). Then rinsed in sterile PBS three times and finally immersed in culture medium.

●  Cell proliferation assay

One day before treatment with the disks cells were allowed to adhere at a density of 5 × 10^4^ cells/well for MDBK cells and 1 × 10^5^ for HT-29 cells into a 48-well plate. The next day, ethanol sterilized disks were immersed into the well containing the cell monolayer and incubated for 24 and 48 h. For cell proliferation assay a MTS ([3-(4,5-dimethylthiazol-2-yl)-5-(3-carboxymethoxyphenyl)-2-(4-sulfophenyl)-2*H*-tetrazolium, inner salt])colorimetric method (CellTiter 96^®^ Aqueous Non-Radioactive Cell Proliferation Assay, Promega, Madison, WI, USA) was used [68]. Briefly, 240 µL of specific cell line culture medium and methyl tetrazolium sulphate was added to each well until the reaction developed. After 15–45 min of incubation, 100 µL of the culture solution was transferred to a 96-well plate clear bottom (Nunc, Thermo Scientific, Waltham, MA, USA) and optical density was measured by a microplate reader (Mithras LB 940 DLReady) (Berthold Technologies, Bad Wildbad, Germany) at 450 nm. Cell viability was expressed as percent of control (untreated cells). 

●  Microscopy method

The morphology of HT-29 and MDBK cells after incubation with the disks for 24 and 48 h were monitored using an automated platform for live cell imaging. The probes were scanned using TissueFAXSiPlus brightfileld imaging system (TissueGnostics GmbH, Vienna, Austria) with a 20× objective of a Zeiss AxioImager.Z1 inverted microscope and fields of view were generated.

## 4. Conclusions

Newly synthesized hydrogel nanocomposites based on organically (unmodified and modified) montmorillonite were prepared and their properties were tested. The experiments derived from this study prove for the first time the capacity of hydrophobic modified commercial clay to slow down the release process of water from hydrogel network and moreover, the enhanced mechanical behavior of the final materials. These results are strongly related to the organomodified structure of clay leading to the intercalated structure of the final nanocomposites. In vitro studies revealed a good biocompatibility of PMMA-clay based novel structures. 

Altogether, the newly synthesized nanocomposite hydrogels could be recommended for situations where controlled release of the drug is required and hydrogel based material should reach to a specific target totally unaltered. Future perspectives include the synthesis of interpenetrated hydrogel matrices using a natural polysaccharide. The method will connect the biopolymer with pH responsive PMAA to design new semi-interpenetrated networks with encapsulated amphiphilic nanoclays to render tunable and efficient advanced materials exhibiting enhanced mechanical strength, improved stability and adjustable responsive properties, for co-delivery of anti-cancer drugs in gastrointestinal tract. The pH change from the stomach to the intestine could be used as a trigger for releasing encapsulated therapeutic agents from pH-responsive semi interpenetrated hydrogel networks-newly functionalized clay nanocomposites. In order to evaluate the influence of the novel functionalized clay system on the sustained release of the anticancer drugs, the final complex systems are foreseen to be investigated in vitro and in vivo. Biodistribution profiles, pharmacokinetic profile and antitumor activity of the drug loaded clay nanocomposite vehicle will be followed in comparison with the neat hydrogel network to prove its enhanced efficacy.

## Figures and Tables

**Table 1:** XRD data obtained for the hydrogel nanocomposites.

Sample	2 Theta (Å)		d-Spacing (nm)	
	(001)	(002)	(001)	(002)
**Cl 93A**	3.28	6.55	26.88	13.48
**PMAA**	-	-	-	-
**PMAA-Cl 93A**	2.99	6.39	29.48	13.83
**PMAA-Cl 93A-C8**	3.12	6.40	28.22	13.80
**PMAA-Cl 93A-C18**	2.99	6.43	29.50	13.74

**Table 2:** TGA/DTG and DMA results for the obtained hydrogel samples.

Sample	TGA/DTG						DMA	
	Weight Loss Intervals (%)			Decomposition Temperatures (°C)		Residue 700 °C (%)	T_g1_ (°C)	T_g2_ (°C)
	0–120 °C	120 °C–300 °C	300 °C–700 °C	T_1_	T_2_			
**A**	8.28	10.63	71.43	230	420	9.59	85.15	226.30
**B**	6.46	10.88	70.88	229	421	11.43	96.52	227.46
**C**	6.96	10.30	71.26	231	380/419	11.48	94.36	233.27
**D**	6.71	10.10	70.47	230	381/410	12.73	110.81	231.56
